# Combating Antibiotic Resistance: Mechanisms, Multidrug-Resistant Pathogens, and Novel Therapeutic Approaches: An Updated Review

**DOI:** 10.3390/ph18030402

**Published:** 2025-03-12

**Authors:** Mostafa E. Elshobary, Nadia K. Badawy, Yara Ashraf, Asmaa A. Zatioun, Hagar H. Masriya, Mohamed M. Ammar, Nourhan A. Mohamed, Sohaila Mourad, Abdelrahman M. Assy

**Affiliations:** 1 Botany and Microbiology Department, Faculty of Science, Tanta University, Tanta 31527, Egypt; 2 Aquaculture Research, Alfred Wegener Institute (AWI)—Helmholtz Centre for Polar and Marine Research, Am Handelshafen, 27570 Bremerhaven, Germany; 3 Applied and Analytical Microbiology Department, Faculty of Science, Ain Shams University, Cairo 11566, Egypt; 4 Microbiology and Chemistry Department, Faculty of Science, Damanhour University, Damanhour 22514, Egypt; 5 Microbiology and Biochemistry Program, Faculty of Science, Benha University-Obour Campus, Benha 13518, Egypt; 6 Faculty of Pharmacy, Sinai University, Ismailia 41636, Egypt; 7 Faculty of Medicine, Alexandria University, Alexandria 21526, Egypt

**Keywords:** multidrug-resistant bacteria, efflux pumps, quorum sensing, probiotics, antimicrobial peptides, venoms, nanobiotics, CRISPR-Cas, immunotherapy, photodynamic therapy

## Abstract

The escalating global health crisis of antibiotic resistance, driven by the rapid emergence of multidrug-resistant (MDR) bacterial pathogens, necessitates urgent and innovative countermeasures. This review comprehensively examines the diverse mechanisms employed by bacteria to evade antibiotic action, including alterations in cell membrane permeability, efflux pump overexpression, biofilm formation, target site modifications, and the enzymatic degradation of antibiotics. Specific focus is given to membrane transport systems such as ATP-binding cassette (ABC) transporters, resistance–nodulation–division (RND) efflux pumps, major facilitator superfamily (MFS) transporters, multidrug and toxic compound extrusion (MATE) systems, small multidrug resistance (SMR) families, and proteobacterial antimicrobial compound efflux (PACE) families. Additionally, the review explores the global burden of MDR pathogens and evaluates emerging therapeutic strategies, including quorum quenching (QQ), probiotics, postbiotics, synbiotics, antimicrobial peptides (AMPs), stem cell applications, immunotherapy, antibacterial photodynamic therapy (aPDT), and bacteriophage. Furthermore, this review discusses novel antimicrobial agents, such as animal-venom-derived compounds and nanobiotics, as promising alternatives to conventional antibiotics. The interplay between clustered regularly interspaced short palindromic repeats (CRISPR) and CRISPR-associated proteins (Cas) in bacterial adaptive immunity is analyzed, revealing opportunities for targeted genetic interventions. By synthesizing current advancements and emerging strategies, this review underscores the necessity of interdisciplinary collaboration among biomedical scientists, researchers, and the pharmaceutical industry to drive the development of novel antibacterial agents. Ultimately, this comprehensive analysis provides a roadmap for future research, emphasizing the urgent need for sustainable and cooperative approaches to combat antibiotic resistance and safeguard global health.

## 1. Introduction

Antibiotic resistance has emerged as a grave global public health concern, primarily attributed to the indiscriminate use of antibiotics in healthcare, veterinary care, aquaculture, and agriculture [1,2]. This trend has catalyzed the proliferation of antimicrobial-resistant (AMR) bacteria, challenging our ability to combat infectious diseases [2,3]. Despite efforts to develop new pharmaceutical solutions, infections caused by pathogens like *Pseudomonas aeruginosa* and multidrug-resistant Enterobacteriaceae have become increasingly impervious to existing antibiotics [4]. The World Health Organization ranks antibiotic resistance among the top 10 threats to global health [5]. This crisis underscores the urgent need for responsible antibiotic stewardship, novel therapeutic strategies, and continued research to curb the spread of AMR. In vitro antimicrobial susceptibility testing has been crucial in assessing resistance levels, with multidrug-resistant microbes emerging as a central concern [5].

Antibiotic resistance is a complex natural process influenced by environmental conditions, microbial community density, and widespread antibiotic use in healthcare, agriculture, and food production [6]. Antibiotic-resistant pathogens spread resistance genes through multiple horizontal gene transfer (*HGT*) mechanisms, including transformation, conjugation, and transduction. Additionally, outer membrane vesicles (OMVs) serve as vehicles for genetic exchange, further enhancing the dissemination of resistance traits among bacterial populations [7]. The discovery of penicillin in 1928 marked a turning point in drug development, leading to the current annual production of about 100,000 tonnes of antibiotics [8]. However, many bacterial species have evolved resistance to multiple drugs, resulting in multidrug resistance [9]. This evolution from the initial breakthrough of penicillin to the current crisis of widespread resistance highlights the urgent need for innovative strategies to combat antimicrobial resistance and safeguard public health. The adaptive nature of bacteria and the continued pressure from antibiotic use underscore the complexity of this challenge and the importance of developing new approaches to maintain effective treatments for infectious diseases.

Multidrug resistance (MDR) typically refers to bacteria resistant to three or more distinct antibiotic classes [10,11]. The human toll of antimicrobial resistance (AMR) is severe, causing over 700,000 deaths annually worldwide, with projections reaching 10 million by 2050 [5]. The economic impact is equally staggering, with AMR projected to cost USD 100 trillion annually by 2050 [12]. Global estimates indicate that antibiotic resistance poses a severe economic burden, particularly in low- and middle-income countries, due to extended hospital stays, higher treatment costs, and increased mortality. The total global cost of antimicrobial resistance is difficult to quantify precisely, but studies suggest that antibiotic resistance could cause up to 10 million deaths annually by 2050, significantly impacting healthcare expenses worldwide [13]. Additionally, the economic cost per antibiotic consumed varies by drug class and income level, underscoring the need for comprehensive policy interventions [14]. For a more in-depth global analysis, further data integration across multiple healthcare systems is necessary [15]. These alarming statistics underscore the urgent need for decisive action in combating antibiotic resistance to mitigate both the devastating loss of human life and the enormous economic burden on global healthcare systems.

The development of new antibiotics faces significant scientific, economic, and regulatory challenges that further complicate the fight against antimicrobial resistance [16]. Pharmaceutical companies have increasingly withdrawn from antibiotic research due to low financial returns and high development costs, with estimates suggesting that bringing a new antibiotic to market requires approximately USD 1 billion and 10–15 years of research [17,18]. The complex regulatory landscape, stringent safety requirements, and limited market incentives create substantial disincentives for pharmaceutical innovation [19,20]. Moreover, the rapid emergence of bacterial resistance mechanisms means that newly developed antibiotics quickly become less effective, further diminishing the economic viability of antibiotic research [21]. Scientifically, the complexity of bacterial adaptation, coupled with the increasing sophistication of resistance mechanisms, makes discovering novel antimicrobial compounds extremely challenging [22]. Traditional screening methods have yielded diminishing returns, necessitating more advanced approaches such as computational drug design, metagenomic exploration, and interdisciplinary strategies that integrate microbiology, genetics, and advanced computational techniques [23,24].

This comprehensive review examines the complex mechanisms of antibiotic resistance in multidrug-resistant bacteria, focusing on four key processes: limiting drug absorption, altering drug targets, drug inactivation, and active drug efflux. These mechanisms vary based on bacterial cell morphology and structure, affecting both acquired and innate resistance. This review explores strategies to mitigate resistance evolution and enhance antimicrobial efficacy, highlighting promising approaches such as quorum-sensing inhibitors (QSIs) and quorum quenchers (QQs). Novel findings include the susceptibility of some resistant strains to scorpion venom, the potential of DsbA protein blockade, and the efficacy of antibacterial photodynamic therapy against resistant *Staphylococcus aureus*. This review also discusses innovative approaches beyond traditional pharmacology, including using mesenchymal stem cells, immunotherapeutic cells, antimicrobial peptides, and probiotics, with the latter showing potential in reducing antibiotic resistance genes in the gastrointestinal environment.

## 2. Types and Resistance Mechanisms of Bacteria to Antibiotics

The resistance mechanisms of bacteria to antibiotics can be comprehensively categorized into three primary types: intrinsic, acquired, and adaptive resistance. Each type represents a distinct strategy employed by bacteria to survive antibiotic exposure.

### 2.1. Intrinsic Resistance

Intrinsic resistance is a natural and inherent characteristic of bacterial species, rooted in fundamental chromosomal elements. This type of resistance involves structural barriers like lipopolysaccharides (LPS) in Gram-negative bacteria, inherent antimicrobial resistance genes (ARGs), and naturally occurring defense mechanisms. Key features include beta-lactamase enzymes, efflux pumps that expel antimicrobial agents, and structural variations that prevent antibiotic penetration [25].

### 2.2. Acquired Resistance

Acquired resistance involves the horizontal transfer of genetic material between bacterial species, enabling a dynamic spread of resistance traits. This mechanism allows bacteria to acquire resistance through processes such as conjugation, transformation, and transduction. The potential for cross-species transmission of resistance genes makes this type of resistance particularly concerning, as it can rapidly extend antibiotic resistance beyond individual bacterial populations [25].

### 2.3. Adaptive Resistance

Adaptive resistance emerges from environmental pressures within microbial niches, characterized by dynamic cellular responses to antibiotic stress. These adaptive mechanisms involve modifications in cell permeability, biofilm formation, and metabolic adaptations that allow bacteria to survive in challenging environments. The ability to quickly modify cellular strategies provides bacteria with a significant survival advantage against antimicrobial treatments [25]. The specific mechanisms driving heightened resistances are multifaceted, including biofilm formation, the alteration of antibiotic binding sites, the production of enzyme-based antibiotic deactivation, reduced membrane permeability, and the active efflux of antimicrobial agents. When these resistance mechanisms converge and interact, they can synergistically contribute to enhanced drug resistance, significantly complicating treatment strategies [25]. Table 1 summarizes and provides a multidimensional view of bacterial resistance mechanisms.

## 3. Mechanisms of Antibiotic Resistance in Bacteria

**Figure 1:**
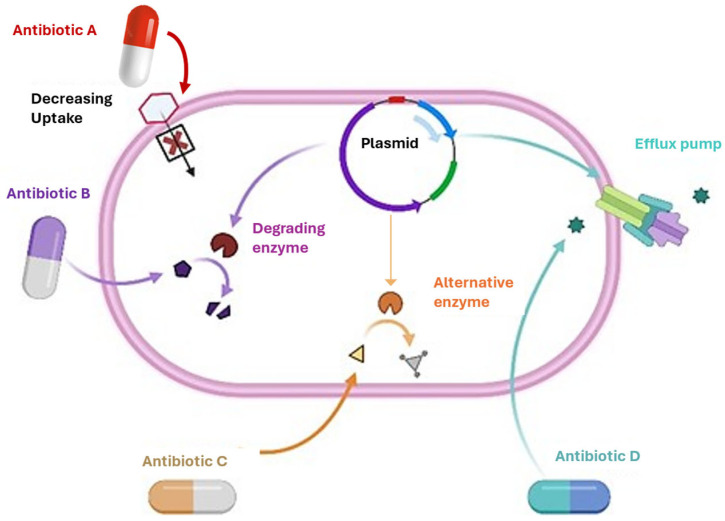
Mechanisms of antibiotic resistance in bacteria include decreased uptake (Antibiotic A, red), enzyme degradation (Antibiotic B, purple), resistance mutation (Antibiotic C, orange), and efflux pumps (Antibiotic D, blue-green).

Bacterial antibiotic resistance mechanisms include enzymatic modification or the inactivation of the antibiotic, the reduced binding affinity of the antibiotic to its bacterial molecular targets, horizontal gene transfer via plasmids carrying multidrug resistance (MDR)-associated genes between species, changes in the permeability of the bacterial cell surface, the overexpression of efflux pumps capable of recognizing and expelling a wide range of antibiotics with diverse mechanisms of action, and genetic variations such as polymorphisms or insertions in DNA sequences encoding transcriptional regulators (Figure 1) [33].

Efflux pumps are integral membrane transporters that play a critical role in bacterial pathogenesis, metabolism, and multidrug resistance [33]. By actively reducing the intracellular concentrations of external agents, such as antibiotics, disinfectants, and detergents, these pumps prevent such substances from reaching their intended biological targets. As a result, efflux transporters have become promising targets for the development of new inhibitors to combat MDR-associated infectious diseases [34].

### 3.1. Membrane Transport Systems in Antibiotic Resistance

**Figure 2:**
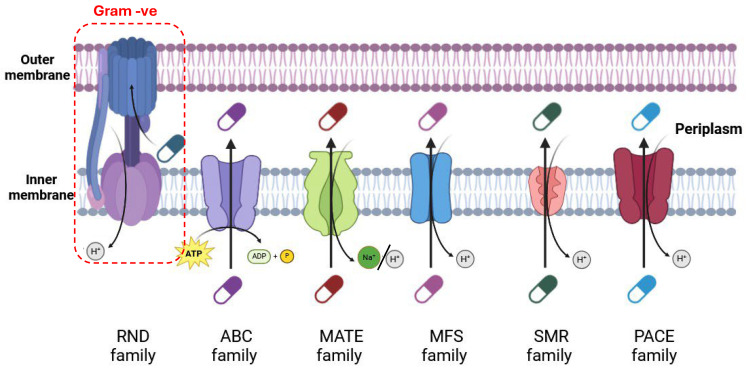
Schematic representation of the major multidrug efflux pump families. RND (tripartite system, proton-gradient-dependent, exports large molecules), ABC (ATP-dependent, found in all domains of life), MATE (sodium/proton-gradient-dependent, single-component system), MFS (proton-gradient-dependent, single-component, ubiquitous), SMR (proton-gradient-dependent, smallest transporter, functions as a dimer), and PACE (proton-gradient-dependent, specific to Proteobacteria, resists biocides and dyes). Each transporter is depicted with distinct shapes and colors, indicating its energy source and substrate transport mechanism. A generic antibiotic is symbolized as a pill.

Membrane transport systems play a crucial role in bacterial antibiotic resistance by actively expelling antimicrobial compounds from bacterial cells, thereby reducing their intracellular concentration to levels below the effective therapeutic threshold. These systems encompass several distinct superfamilies, each characterized by unique structural and functional attributes that contribute to antimicrobial resistance. Based on their sequence homology, substrate specificity, structural features, and energy sources, efflux transporters are categorized into five major superfamilies: the multidrug and toxic compound extrusion (MATE) superfamily, small multidrug resistance (SMR) superfamily, ATP-binding cassette (ABC) superfamily, proteobacterial antimicrobial compound efflux (PACE) superfamily, and resistance nodulation and cell division (RND) superfamily, along with the major facilitator superfamily (MFS) [34] (Figure 2).

#### 3.1.1. ATP-Driven Efflux Pumps (ABC)

ATP-binding cassette (ABC) transporters are a major protein superfamily in bacteria, facilitating both nutrient uptake and xenobiotic efflux. These transporters consist of two transmembrane domains (TMDs) that form the substrate transport pathway and two nucleotide-binding domains (NBDs) that drive efflux via ATP hydrolysis. In Gram-negative bacteria, periplasmic binding proteins often aid in substrate recognition and transport initiation [35].

ABC transporters play a dual role in antibiotic resistance and virulence. Export systems actively pump out antibiotics, as seen in the MacAB-TolC system. A tripartite efflux system in *Escherichia coli* consists of MacA (membrane fusion protein), MacB (ATP-binding cassette transporter), and TolC (outer membrane channel), which contributes to macrolide resistance and virulence factor secretion in *Enterobacteriaceae*. Studies in *Serratia marcescens* have demonstrated that deleting macAB genes increases bacterial susceptibility to aminoglycosides and cationic antimicrobial peptides (CAPs), though with varying effects on different antibiotics [36].

The expression of ABC transporters is tightly regulated by substrate-responsive transcriptional regulators and two-component regulatory systems. Current research aims to develop ABC transporter inhibitors as antibiotic adjuvants, though challenges remain due to the redundancy and complex regulation of bacterial efflux systems [37].

#### 3.1.2. Major Facilitator Superfamily (MFS)

The major facilitator superfamily (MFS) is the largest and most extensively characterized group of transmembrane secondary transport proteins in both prokaryotic and eukaryotic systems. MFS transporters mediate substrate translocation via passive transport mechanisms, including uniport (transport of a single substrate) and secondary active transport mechanisms, such as symport (co-transport of a substrate with a coupling ion in the same direction) and antiport (exchange of substrates in opposite directions). The substrates transported by MFS proteins are remarkably diverse, encompassing sugars, amino acids, metabolic intermediates, ions, and antimicrobials [38].

A chromosomally encoded MFS multidrug efflux pump, designated *SA09310* gene, has been identified in Staphylococcus aureus. This 43-kDa protein features 12 transmembrane helices (TMHs), a hallmark structural characteristic of MFS transporters. SA09310 contains conserved amino acid sequence motifs specific to MFS transporters, confirming its classification within this family. Functionally, *SA09310* has been implicated in resistance to a broad spectrum of antibiotics, including aminoglycosides, tetracyclines, macrolides, and chloramphenicol. Experimental evidence indicates that SA09310 mediates antibiotic resistance by actively exporting intracellular tetracycline from bacterial cells into the extracellular environment. This efflux mechanism reduces intracellular antibiotic concentrations, thereby preventing the drug from reaching its biological targets [39].

*SCO4121*, another MFS transporter gene, has been identified in *Streptomyces coelicolor* and characterized for its role in multidrug resistance (MDR). Overexpression studies of *SCO4121*, both in native and heterologous host systems, demonstrate its capacity to confer resistance to a variety of antibiotics, including ciprofloxacin and chloramphenicol. Conversely, the deletion of the *SCO4121* gene significantly increases bacterial susceptibility to these drugs, highlighting its function as a major efflux transporter. In addition to antibiotic resistance, *SCO4121* also confers tolerance to oxidative stress caused by hypochlorous acid (HOCl), a potent oxidizing agent. This dual role underscores the physiological importance of MFS transporters in bacterial survival under diverse environmental stresses. Notably, the expression of *SCO4121* is transcriptionally regulated in a concentration-dependent manner by its substrate drugs, suggesting a sophisticated adaptive response mechanism in wild-type *S. coelicolor* cells [40]. Efflux pumps belonging to the MFS family, such as SA09310 and SCO4121, play a pivotal role in multidrug resistance by reducing intracellular antibiotic concentrations, thus allowing bacteria to survive in the presence of antimicrobial agents. The ability of MFS transporters to efflux diverse substrates highlights their evolutionary adaptation and functional versatility. Consequently, MFS efflux pumps represent promising targets for the development of efflux pump inhibitors (EPIs), which could restore the efficacy of existing antibiotics and help combat MDR in pathogenic bacteria.

#### 3.1.3. Small Multidrug Resistance (SMR) Family

The small multidrug resistance (SMR) family comprises small multidrug-resistant proteins typically ranging from 100 to 140 amino acids in length. These proteins are characterized by their structural organization into four transmembrane α-helices embedded in bacterial membranes. SMR proteins are notable for their ability to confer resistance to a wide range of compounds, particularly quaternary ammonium compounds (QACs) and highly hydrophobic substances, due to their capacity to solubilize these substances in organic solvents.

Functionally, SMR proteins play a critical role in the synthesis and efflux of various lipophilic compounds, including antiseptics, detergents, antibiotics, and other drugs. The SMR family encompasses a diverse group of proteins encoded by genes located on both plasmids and bacterial chromosomes, demonstrating substantial structural and functional heterogeneity. This diversity enables SMR proteins to resist different classes of antibiotics, including β-lactams such as cephalosporins. The genetic basis for this resistance is attributed to the close association between SMR genes and antimicrobial resistance (AMR) genes within bacterial genomes, thereby enhancing the multidrug resistance capabilities of bacterial cells [41].

One noteworthy example of SMR functionality is the *A1S_0710* gene, which encodes an SMR transporter protein in *Acinetobacter baumannii*. The experimental deletion of this gene has revealed a dual role for the encoded protein: its absence results in decreased motility and increased virulence, suggesting that its physiological importance extends beyond antimicrobial resistance [42]. This dual functionality highlights the complex roles SMR proteins play in bacterial survival and pathogenicity.

A prominent member of the SMR family is EmrE, a small multidrug transporter found in *Escherichia coli*. EmrE operates as a proton-driven efflux pump, utilizing the proton motive force (PMF) across the inner bacterial membrane to expel drug molecules. The stoichiometry of proton-to-drug exchange in EmrE is 2:1, wherein two protons are imported into the cell for each drug molecule exported [43]. The functional mechanism of EmrE revolves around a well-characterized binding pocket at the protein’s core, which includes two highly conserved glutamate residues (Glu14) located near the center of the transmembrane domain. This binding pocket interacts with two ligands simultaneously, facilitating their exchange. Recent structural and functional analyses have provided evidence for the existence of a peripheral ligand binding site, which may modulate the transport process without inducing significant conformational changes in the protein. The binding of substrates to this peripheral site has been hypothesized to influence proton translocation indirectly [43].

In addition to Glu14, emerging studies have highlighted the importance of less-characterized residues such as Histidine 110 (His110). Although distant from the core binding site, His110 may play a regulatory role in SMR transport activity and proton coupling. Its precise contribution remains a subject of ongoing investigation, with recent computational and experimental findings suggesting it influences the conformational dynamics of the protein [44].

#### 3.1.4. Multidrug and Toxic Compound Extrusion (MATE) Family

The MATE family, first identified in 2008, was first described in relation to multidrug resistance in bacteria. One of the earliest characterized members, NorM from *Neisseria gonorrhoeae*, was identified as a multidrug efflux pump capable of exporting fluoroquinolones and other toxic compounds [45]. MATE transporters are secondary active transporters, primarily involved in the efflux of cationic substrates, and play a critical role in reducing bacterial susceptibility to a range of antimicrobial agents. These include ethidium bromide, berberine, acriflavin, norfloxacin, and tetraphenylphosphonium, all of which are substrates that can accumulate to toxic levels within bacterial cells if not extruded by these pumps [46].

The expression of MATE transporters is tightly regulated, and alterations in their expression profiles can significantly impact bacterial resistance mechanisms. For example, the overexpression of the MepA transporter in *Staphylococcus aureus* has been shown to confer resistance to tigecycline, an antibiotic frequently used in the treatment of infections caused by methicillin- and vancomycin-resistant *S. aureus* strains [47]. This overexpression enables the bacterium to actively expel tigecycline, thus reducing the intracellular drug concentration and rendering it ineffective in combating the infection [47]. Despite the structural similarities across the MATE superfamily, different members exhibit unique transport mechanisms that reflect their diverse physiological roles. For instance, NorM-VC, a MATE transporter found in *Vibrio cholerae*, and NorM-PS, found in *Pseudomonas stutzeri*, both share comparable structural properties, such as the presence of multiple transmembrane helices forming a central substrate-binding cavity. However, these transporters differ in their energization mechanisms. NorM-PS is driven by H+ electrochemical gradients, where the proton gradient is used to power the extrusion of substrates. In contrast, NorM-VC utilizes both Na+ and H+ gradients to facilitate the transport of substrates, which adds a level of complexity to its transport process and suggests functional divergence within the MATE family [48]. These differences in transport mechanisms reflect the evolutionary adaptability of the MATE family to diverse environmental conditions and substrates. The diverse range of MATE transporters underscores their critical role in bacterial survival, particularly in the context of antibiotic resistance. Targeting these transporters represents a promising strategy for the development of new antimicrobial agents, particularly efflux pump inhibitors (EPIs) that could enhance the efficacy of existing antibiotics by inhibiting MATE-mediated drug extrusion.

#### 3.1.5. Proteobacterial Antimicrobial Compound Efflux (PACE) Family

The proteobacterial antimicrobial compound efflux (PACE) family represents a recently discovered group of bacterial membrane transport proteins that contribute to antimicrobial resistance. First identified in 2013, PACE family transporters have been found predominantly in Proteobacteria, particularly in clinically relevant pathogens such as *Acinetobacter baumannii*, *Klebsiella pneumoniae*, and *Pseudomonas aeruginosa* [49].

PACE family proteins typically consist of about 150–160 amino acid residues and are predicted to have four transmembrane α-helices (TMHs) [50]. Four amino acid residues, glutamic acid, asparagine, alanine, and aspartic acid, are conserved in the family by the alignment of 47 different PACE proteins from various bacterial species. Glutamic acid is found on Transmembrane Helix One. Amino acids are present at the periplasmic membrane boundary of Transmembrane Helix Four, and aspartic acid occurs in the cytoplasmic membrane boundary of Transmembrane Helix Four [49]. These conserved residues are thought to play crucial roles in the protein’s function, and are potentially involved in substrate binding or energy coupling mechanisms. PACE family transporters have been shown to confer resistance to a range of antimicrobial compounds, including chlorhexidine: a widely used biocide in healthcare settings; Acriflavine: an antiseptic agent; Proflavine: another antiseptic compound; and Benzalkonium: a quaternary ammonium compound used as a disinfectant [49]. The prototype member of this family, AceI (*Acinetobacter chlorhexidine* efflux protein I), was first identified in *Acinetobacter baumannii*. AceI has been demonstrated to resist chlorhexidine, a crucial antiseptic in clinical settings [51]. While the exact mechanism of PACE transporters remains under investigation, current evidence suggests they function as proton-dependent efflux pumps. This means they likely use the energy from the proton motive force to actively extrude antimicrobial compounds from the bacterial cell, thereby reducing intracellular drug concentrations and conferring resistance [50].

The discovery of PACE family transporters has significant implications for public health and clinical practice. Their ability to confer resistance to commonly used biocides like chlorhexidine poses challenges for infection control in healthcare settings [52]. Some PACE family members have been associated with multidrug-resistant (MDR) phenotypes in clinically important pathogens, complicating treatment options [49]. Understanding the structure and function of PACE transporters could lead to the development of novel inhibitors, potentially restoring the efficacy of certain antimicrobials [50].

As our knowledge of the PACE family expands, it is becoming increasingly clear that these transporters play a significant role in bacterial antimicrobial resistance. Continued research in this area is crucial for developing effective strategies to combat the growing threat of antibiotic-resistant infections.

#### 3.1.6. Resistance Nodulation Cell Division (RND) Superfamily

The resistance nodulation cell division (RND) superfamily is a large group of proteins found in various organisms, including bacteria, archaea, and some eukaryotes. The RND superfamily proteins play a significant role in antibiotic resistance in bacteria. Many clinically relevant antibiotic resistance mechanisms in Gram-negative bacteria involve RND efflux systems, which contribute to multidrug resistance (MDR) by actively extruding a wide range of antibiotics and other antimicrobial agents from the bacterial cell. The RND superfamily comprises 12 transmembrane helices separated by two large loops, forming asymmetric trimers. The outer loop contains binding sites for exported ligands, while the transmembrane domain primarily functions as a channel for protons, providing energy for substrate translocation [53]. These pumps are crucial for bacterial survival, offering a spectrum of functions ranging from pH tolerance to resistance against an array of noxious agents, including toxic natural products, metal ions, bile acids, fatty acids, and host-derived antimicrobial peptides. Due to their diverse roles, including pH tolerance and resistance to various substances such as toxic natural products, metal ions, bile acids, fatty acids, and antimicrobial peptides produced by host cells, RND efflux pumps have garnered significant attention. These pumps are associated with outer membrane proteins (OMPs) and are facilitated by periplasmic adaptor proteins (PAPs). In human-associated bacteria like *Salmonella enterica*, *Escherichia coli*, and *Pseudomonas aeruginosa*, the characteristics of their RND multidrug efflux pumps have been extensively studied compared to other species [54].

In the realm of antimicrobial peptides, CASP (cationic antibiotic-sensitive peptide) emerges as a novel player, demonstrating unique properties in its interaction with bacterial efflux pumps. CASP exhibits a cyclic structure and possesses cationic attributes, allowing it to bind specifically within the periplasmic cleft region of MtrD, a component of the RND pump system. This interaction involves distinct and overlapping amino acid contact sites, distinguishing CASP from other cyclic peptides like colistin and linear cationic antibacterial agents derived from human peptide LL-37 (a human antimicrobial peptide derived from the cathelicidin precursor protein (hCAP18), involved in innate immunity). Although CASP may not sensitize *Neisseria gonorrhoeae* to novobiocin, an antibiotic substrate for the RND pump, it exhibits efficacy against select rod-shaped Gram-negative bacteria, showcasing its potential as a therapeutic agent with a unique mode of action [55].

### 3.2. Reduced Membrane Permeability

One of the fundamental mechanisms of antibiotic resistance involves the bacterial ability to reduce membrane permeability, limiting the intracellular concentration of antibiotics. Gram-negative bacteria, in particular, possess an outer membrane that acts as a selective barrier, preventing the entry of harmful compounds [56]. The primary route for hydrophilic antibiotics, such as β-lactams, fluoroquinolones, and aminoglycosides, is through porin proteins: channel-like structures embedded in the outer membrane. The mutations or downregulation of porins can significantly reduce antibiotic uptake, leading to resistance [57].

For instance, *Pseudomonas aeruginosa* exhibits reduced permeability to carbapenems due to mutations in the OprD porin, which normally facilitates the uptake of imipenem and meropenem [35]. Similarly, *Escherichia coli* strains resistant to cephalosporins have been found to downregulate OmpF and OmpC porins, leading to decreased drug influx. These changes often occur in conjunction with other resistance mechanisms, such as efflux pump overexpression or β-lactamase production, resulting in multidrug resistance.

Additionally, changes in the lipid composition of bacterial membranes can also alter permeability, affecting the diffusion of antibiotics. Studies on *Acinetobacter baumannii* indicate that modifications in lipopolysaccharide (LPS) structure can further enhance resistance by reducing membrane fluidity and increasing charge repulsion against cationic antibiotics [37].

### 3.3. Biofilm Formation

Biofilm formation is a critical survival strategy employed by bacteria to withstand antibiotic treatment and host immune responses. Biofilms are complex three-dimensional microbial communities embedded in a self-produced extracellular polymeric substance (EPS) [58]. The EPS matrix acts as a physical barrier that restricts antibiotic penetration, neutralizes antimicrobial agents, and facilitates the horizontal gene transfer of resistance elements among bacterial cells. Biofilm-associated bacteria exhibit significantly higher tolerance to antibiotics compared to planktonic (free-floating) cells. This resistance can be attributed to the following. Reduced Antibiotic Penetration: The dense biofilm matrix slows down the diffusion of antimicrobial agents, reducing their effectiveness. Altered Metabolic States: Bacteria within biofilms exist in a gradient of metabolic activity, with cells in the deeper layers becoming dormant [4]. These persister cells are highly tolerant to antibiotics that target actively growing bacteria. Quorum Sensing Regulation: Biofilm formation is controlled by quorum sensing, a cell-to-cell signaling mechanism that enhances stress resistance and antibiotic tolerance.

A study on *Staphylococcus aureus* and *Pseudomonas aeruginosa* biofilms revealed that exposure to sublethal antibiotic concentrations can trigger an adaptive response that strengthens biofilm defenses [59]. Moreover, bacterial biofilms are commonly associated with chronic infections, such as cystic fibrosis, catheter-related bloodstream infections, and implant-associated infections, making them a major clinical challenge.

Given the role of biofilms in antibiotic resistance, novel therapeutic strategies are being explored, including biofilm-disrupting agents like quorum-sensing inhibitors, EPS-degrading enzymes, and nanoparticle-based drug delivery systems [60].

### 3.4. Target Site Modifications

Target site modification is a prevalent resistance mechanism where bacteria alter the molecular structures that antibiotics typically bind to, rendering the drugs ineffective. This occurs through genetic mutations, enzymatic modifications, or the acquisition of resistance genes via horizontal gene transfer [37].

Examples of target site modifications include the following. Fluoroquinolone Resistance: Bacteria develop resistance by acquiring mutations in the genes encoding DNA gyrase (gyrA) and topoisomerase IV (parC), the primary targets of fluoroquinolones. These mutations reduce drug binding affinity, leading to decreased susceptibility. β-Lactam Resistance: Changes in penicillin-binding proteins (PBPs) lower the binding affinity of β-lactam antibiotics. Methicillin-resistant *Staphylococcus aureus* (MRSA) expresses PBP2a, a modified enzyme encoded by the mecA gene, which retains transpeptidase activity even in the presence of β-lactams [37]. Macrolide Resistance: The methylation of 23S rRNA by erm genes prevents macrolide antibiotics, such as erythromycin and azithromycin, from binding to the ribosomal subunit, leading to resistance. Such modifications allow bacteria to continue essential cellular processes despite antibiotic presence. This type of resistance is particularly concerning in Acinetobacter baumannii and Klebsiella pneumoniae, where combined target modifications and other resistance mechanisms contribute to extensive drug resistance [35].

### 3.5. Enzymatic Degradation of Antibiotics

Bacteria can neutralize antibiotics through enzymatic degradation, a major mechanism contributing to resistance against β-lactams, aminoglycosides, and chloramphenicol. The production of these enzymes often spreads through plasmids and transposable elements, accelerating resistance dissemination.

Key classes of antibiotic-degrading enzymes include the following. β-Lactamases: These enzymes hydrolyze the β-lactam ring, rendering β-lactam antibiotics ineffective. The extended-spectrum β-lactamases (ESBLs) and carbapenemases (e.g., KPC, NDM, OXA-type) are particularly concerning as they confer resistance to a wide range of β-lactam antibiotics [36]. Aminoglycoside-Modifying Enzymes: Bacteria can enzymatically modify aminoglycosides (e.g., gentamicin, tobramycin) through acetylation, phosphorylation, or adenylation, reducing their binding affinity to ribosomal targets. Chloramphenicol Acetyltransferases (CATs): These enzymes inactivate chloramphenicol by acetylation, preventing it from inhibiting bacterial protein synthesis. Enzymatic degradation is a primary driver of resistance in Enterobacteriaceae and Pseudomonas aeruginosa. Given the increasing prevalence of β-lactamase-mediated resistance, combination therapies using β-lactamase inhibitors (e.g., clavulanic acid, avibactam) have been developed to restore β-lactam efficacy. However, the emergence of metallo-β-lactamases (MBLs), which are resistant to current inhibitors, presents an ongoing challenge [61].

## 4. Strategies to Combat Antibiotic Resistance

Antibiotic resistance remains a critical challenge in global healthcare, threatening the effectiveness of treatments and increasing the burden of resistant infections. While no single approach can completely eliminate this problem, various strategies can help mitigate its spread and impact. Antibiotic overuse is a major driver of resistance evolution, as epidemiological studies have shown a direct link between consumption and the emergence of resistant strains [4]. Resistance genes can transfer between bacterial species via horizontal gene transfer or arise naturally through mutations, which are processes exacerbated by inappropriate prescribing practices and suboptimal dosing [7]. These factors not only enhance pathogen virulence, but also accelerate the dissemination of resistance determinants.

Addressing these challenges requires a multifaceted approach that includes improving antibiotic stewardship, enhancing infection prevention measures, and advancing alternative treatments such as phage therapy and immunotherapies. Public awareness campaigns and education on the prudent use of antibiotics also play a vital role in mitigating resistance. The declining efficacy of conventional antibiotics has necessitated the investigation of alternative antimicrobial approaches. This section examines emerging interventions for combating antibiotic resistance, including quorum quenching mechanisms that disrupt bacterial communication networks, microbial-based therapies, stem cell applications, immunotherapeutic modalities, photodynamic antimicrobial techniques, and CRISPR-Cas systems and their relationship to resistance mechanisms, bacteriophage therapy, and bioactive compounds derived from animal venoms.

### 4.1. Quorum Quenching (QQ): Targeting Bacterial Communication

Quorum sensing (QS) is a bacterial cell-to-cell communication mechanism that regulates group behaviors, including biofilm formation, virulence factor production, and antibiotic resistance [62]. QS involves the synthesis, accumulation, and recognition of signaling molecules, which trigger changes in gene expression once a critical bacterial density is reached [63]. These coordinated processes enable bacteria to function collectively, enhancing their pathogenicity and resistance [64]. QS systems vary across bacterial types: Gram-negative bacteria rely on acyl-homoserine lactones (AHLs), Gram-positive bacteria use autoinducing peptides, and both groups can utilize autoinducer-2 molecules as a universal signaling system [65,66].

**Figure 3:**
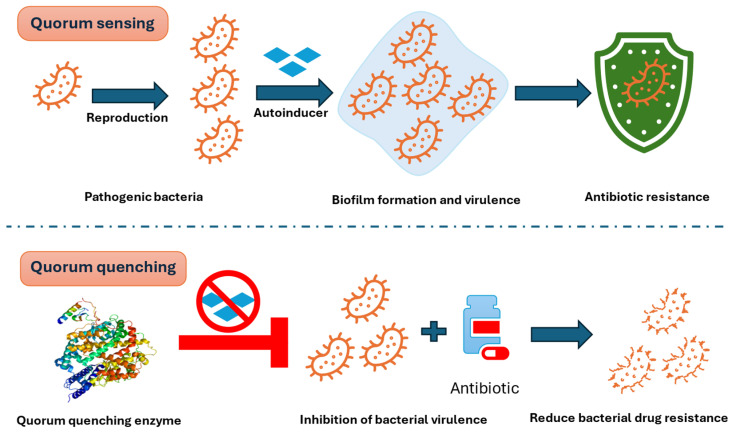
Mechanism diagram of the quorum quenching inhibition of the quorum sensing of pathogenic bacteria.

Quorum quenching (QQ) involves the inhibition of quorum sensing (QS) through chemical or enzymatic means, aiming to mitigate behaviors regulated by QS and thereby reduce bacterial pathogenicity (Figure 3). This approach also enhances the efficacy of antibiotic and phage treatments [67]. QS communication can be disrupted through the enzymatic breakdown of Acyl-homoserine lacton (AHL), a signal molecule in Gram-negative bacteria, by lactonases, acylases, and oxidoreductases. Alternatively, it can be achieved by employing small structural compounds that prevent the QS signal molecule from binding to its regulatory protein [68]. The high-density colony population can produce a sufficient amount of small-molecule signals to activate a variety of downstream cellular processes, including virulence and drug resistance mechanisms, and resist antibiotics [69]. Quorum sensing (QS) induces changes in several aspects to minimize bacterial antibiotic resistance. This includes suppressing, deactivating, and disrupting QS signaling molecule production [65]. Bacterial communities continuously produce extracellular polymeric substances (EPS), enhancing the durability of biofilm structures. QS quenching can disrupt the production and maintenance of the EPS matrix, leading to a loss of biofilm structural integrity and increased bacterial drug resistance [70]. Additionally, QS systems can alter the composition of cell membranes and suppress the expression of virulence, efflux pump, and antioxidant genes.

Improved membrane permeability and the suppression of efflux pump mechanisms enhance the ability of antibiotics to penetrate bacterial cells. Natural phytochemical constituents derived from *Sambucus nigra* and *Matricaria chamomilla* demonstrate significant quorum quenching (QQ) activity through specific molecular interference with bacterial communication systems [71]. These compounds interact with quorum sensing (QS) machinery by competitively binding to receptor proteins or degrading signaling molecules such as N-acyl homoserine lactones (AHLs) in Gram-negative bacteria and autoinducing peptides (AIPs) in Gram-positive species [72]. For instance, vanillin and trans-cinnamaldehyde from cinnamon bark directly inhibit signal molecule recognition and subsequent virulence gene transcription [73]. Similarly, salicylic acid disrupts the las and rhl QS systems in *Pseudomonas aeruginosa* by downregulating the production of signaling molecules and interfering with regulatory protein function [74]. These QQ mechanisms ultimately attenuate biofilm formation, virulence factor production, and pathogenicity without imposing selective pressure that would promote resistance development. The molecular specificity of these phytochemicals for QS components presents a promising therapeutic approach that targets bacterial virulence rather than viability, potentially circumventing conventional resistance mechanisms.

### 4.2. Probiotics, Postbiotics, Prebiotics, and Synbiotics

**Figure 4:**
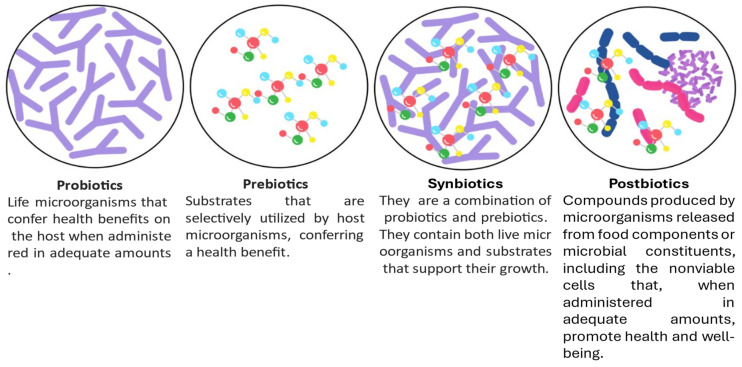
Definition of probiotics, prebiotics, synbiotics, and postbiotics.

Microbial resistance to antibiotics has become a critical global health challenge, significantly complicating medical treatments [75]. In light of this issue, strategies that enhance the host’s immune system, particularly through the modulation of the gut and body microbiota, have gained increasing attention. Probiotics, postbiotics, and synbiotics represent promising approaches to restore microbial balance, alleviate infections, and reduce over-reliance on antibiotics [76] (Figure 4) (Table 2). These interventions are integral to maintaining or restoring health by targeting the host’s microbiota, thereby providing alternative therapeutic strategies.

Probiotics, which are live microorganisms, offer health benefits when administered in appropriate amounts. These beneficial bacteria can modulate the gut microbiota, enhance immune responses, and contribute to the overall well-being of the host [77]. Probiotics perform their beneficial effects through various mechanisms, including competitive exclusion, the production of antimicrobial substances, and the modulation of immune responses. They are capable of producing a wide range of bioactive metabolites, such as lactic and acetic acids, ethanol, carbon dioxide, hydrogen peroxide, and bacteriocins, all of which contribute to their antibacterial and immune-modulatory properties [75]. Furthermore, for probiotics to effectively colonize the host and exert beneficial effects, they must be resistant to digestive fluids, bile salts, sodium chloride, and acidic environments, ensuring their survival during gastrointestinal transit [78]. Probiotics must also meet rigorous safety and efficacy standards, supported by clinical evidence, to confirm their health benefits [79].

Postbiotics are the biologically active metabolites or non-viable bacterial products generated by probiotics during fermentation in the host. These metabolites enhance the efficacy of probiotics by exerting their biological effects, such as antimicrobial, anti-inflammatory, and immunomodulatory actions [80]. Postbiotics include a wide range of compounds, including cell wall fragments, proteins, peptides, and organic acids, which have been shown to reduce the risk of infections and improve gut health. Unlike probiotics, postbiotics do not require live microorganisms, making them a more stable and safer alternative in certain clinical settings. Their effectiveness stems from their ability to modulate the immune system, inhibit pathogen growth, and restore microbial balance.

Prebiotics play a crucial role in promoting the growth and activity of beneficial bacteria in the host’s gut. These indigestible food components support microbial health by providing substrates for beneficial microorganisms, thus enhancing their activity and facilitating the production of bioactive metabolites [81].

Synbiotics refer to the combination of prebiotics and probiotics, designed to provide synergistic effects by enhancing the survival and activity of beneficial microorganisms while simultaneously promoting their growth through prebiotic substrates. This combined approach maximizes the therapeutic potential of both probiotics and prebiotics, supporting a healthy gut microbiota, improving nutrient absorption, and enhancing the immune response [82]. Synbiotics are particularly effective in addressing various gastrointestinal and systemic conditions, as they not only replenish beneficial microbes, but also create an environment conducive to their long-term survival and activity [83].

Probiotics and synbiotics offer promising therapeutic alternatives to antibiotics, particularly in addressing chronic conditions such as gastrointestinal disorders, metabolic diseases, and infections associated with antimicrobial resistance. By competing for binding sites, nutrients, and space, probiotics prevent pathogenic bacteria from colonizing the gut, thus reducing the likelihood of infections. Furthermore, probiotics can directly disrupt bacterial biofilms, which are complex microbial communities that contribute to chronic infections and resistance to antibiotics [84]. By interfering with biofilm formation and limiting horizontal gene transfer, probiotics help reduce the spread of antibiotic resistance.

The immune-modulatory effects of probiotics and synbiotics also play a crucial role in maintaining host health. Probiotics stimulate the production of antimicrobial peptides, modulate gut-associated lymphoid tissue (GALT), and promote a balanced inflammatory response, which can help prevent excessive immune activation associated with conditions like inflammatory bowel disease (IBD) and allergies [85].

In summary, probiotics, postbiotics, and synbiotics offer valuable alternatives to traditional antibiotic therapies. These biologically active agents not only enhance immune function and restore microbial balance, but also help mitigate the risks associated with antibiotic resistance. Further research is essential to fully elucidate their mechanisms of action, therapeutic potentials, and specific clinical applications.

**Table 2:** Some examples of postbiotics, probiotics, and synbiotics and their action against antimicrobial resistance bacteria.

Prebiotics, Postbiotics, Probiotics, and Synbiotics	Action	AMR Bacteria	References
Prebiotics			
Fructooligosaccharides (FOS), Inulin, Galactooligosaccharides (GOS), Polydextrose	Stimulate beneficial microbial growth, improve gut health, enhance immunity, and reduce pathogen colonization	*Escherichia coli*, *Clostridium difficile*, *Bacteroides fragilis*, *Streptococcus mutans*	[86,87]
Postbiotics			
Short-chain fatty acids, enzymes, vitamins, extracellular polysaccharides, cell wall fragments and bacterial lysates	Positively affect immunity, are anti-inflammatory, antibacterial, anti-carcinogenic, and antibiofilm	*Fusobacterium*, *Clostridioides difficile*, *Escherichia coli*, *Streptococcus mutans*, *Porphyromonas gingivalis*, *Tannerella forsythia*, *Prevotella loescheii*, and *Salmonella* spp.	[88]
Probiotics			
*Lactobacillus rhamnosus* GG and *Lactobacillus acidophilus* produce bacteriocins such asrhamnosin and lactocin	Are anticancer and alleviate intestinal damage, mucositis, and antibiofilm	*Clostridium difficile*, *Escherichia coli*, and *Pseudomonas aeruginosa*	[89]
Bifidobacterium produce bifidocin and lactic acid	Regulate inflammation and antimicrobial peptides, reduce antibiotic overuse, and improve gut health	*Pseudomonas aeruginosa* and *Clostridium difficile*	[90]
*Leuconostoc mesenteroides* MJM60376 and *Leuconostoc mesenteroides* LVBH107	Antibiofilm and antimicrobial activities	*Porphyromonas gingivalis* and *Streptococcus mutans* KCTC3065	[91]
Synbiotic			
*Bifidobacterium lactis* BL-99 with Fructooligosaccharide (FOS)	Regulate intestinal microbiota	*Bilophila*, *Escherichia*, and *Shigella*	[92]
*Lactobacillus paracasei* VL8 and Mannan oligosaccharide (MOS)	Positive role in foodborne pathogens	*Salmonella Typhimurium*	[93]
*Lactobacillus rhamnosus* GG and Fructooligosaccharide (FOS) Produces Bacteriocins like rhamnosin	Coordinating gut microbiota	*Shigella sonnei*, *Salmonella typhimurium*, *Klebsiella pneumoniae*, and *Clostridioides difficile*	[94]
*Streptococcus thermophiles* and Xylooligosaccharides (XOS)	Positive role in colorectal cancer	*Helicobacter hepaticus*, *Helicobacter pylori*, *Escherichia coli*, *Enterococcus faecalis*, and *Streptococcus bovis*	[95]
*Streptococcus thermophiles* and Fructooligosaccharide (FOS)	Overcoming diarrhea	*Clostridioides difficile*	[96]

### 4.3. Stem Cells

**Figure 5:**
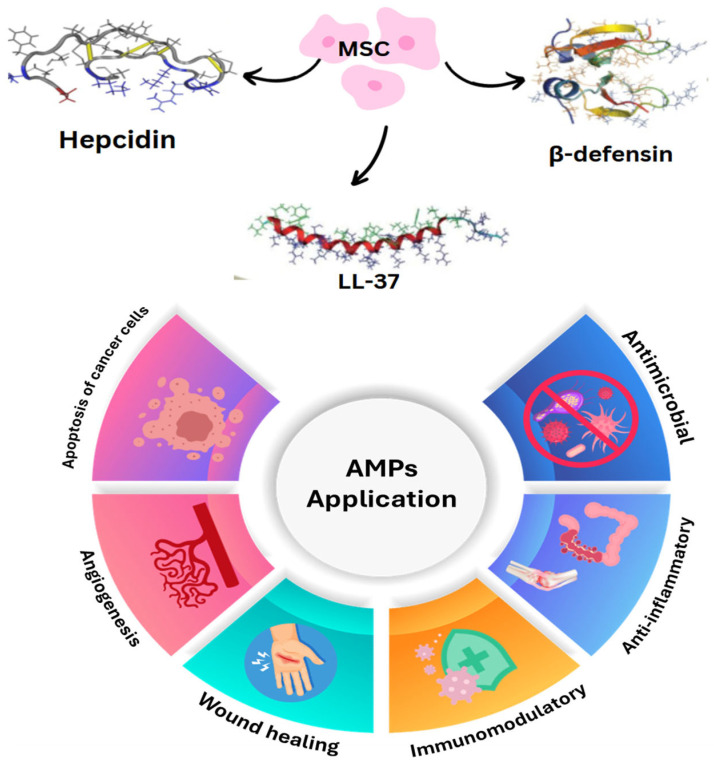
MSCs produce antimicrobial peptides (AMPs), including hepcidin, LL-37, and β-defensin, and their applications are shown.

Stem cells, particularly mesenchymal stem cells (MSCs), have emerged as promising agents in combating antimicrobial resistance (AMR). These cells possess innate antimicrobial properties and can be sourced from various origins, including induced pluripotent stem cells (iPSCs), adult tissues, fetal membranes, and embryonic stem cells (ESCs). The mechanisms by which MSCs combat antimicrobial resistance are twofold. First, they directly interact with cellular systems to inhibit bacterial growth, and second, they enhance the host’s innate immune system’s capacity to fight pathogens. This is achieved through multiple pathways, including the secretion of antimicrobial peptides and the ability to modify the surrounding cellular environment to impede microbial proliferation. Recent studies highlight MSCs’ role in eliminating AMR bacteria through biofilm degradation and the secretion of AMPs such as β-defensin-2, LL-37, and hepcidin [97] (Figure 5). These peptides disrupt bacterial cell walls and alter the microenvironment to inhibit bacterial growth. Furthermore, MSCs work synergistically with antibiotics to enhance their effectiveness in degrading biofilms, a key factor in bacterial resistance [97].

In wound healing and immune regulation, MSCs promote CXCR4 activity, a receptor essential for hematopoiesis and immune response modulation. CXCR4, a G-protein-coupled receptor (GPCR), interacts with its ligand CXCL12 to mediate immune cell activation. Observations suggest that increased CXCR4 expression enhances the extrafollicular B-cell response and early B-cell activation, further amplifying the antimicrobial effects [98,99]. This interplay of MSCs, AMPs, and CXCR4 forms a multi-pronged approach to tackling AMR by simultaneously boosting host immunity, disrupting bacterial biofilms, and enhancing antibiotic efficacy. The integration of MSC-based therapies with AMPs has shown great potential in developing innovative treatments for AMR infections (Figure 5).

AMPs represent a novel class of therapeutics against bacterial infections, offering an alternative to conventional antibiotics. Often referred to as peptide antibiotics, AMPs are small molecules (7–50 amino acid residues, <10 kDa) that are positively charged (+2 to +9). These peptides are integral to the innate immune system, targeting bacteria, fungi, and parasites [98]. AMPs are primarily stored in polymorphonuclear neutrophils (PMNs) and macrophages (MQ) and are produced by various cell types, including epithelial cells and MSCs. Among the well-studied AMPs is LL-37, identified in 1995 as a cathelicidin peptide derived from human bone marrow [100]. LL-37 exhibits potent antibacterial activity and plays a key role in host defense by promoting angiogenesis and tissue regeneration, attracting and activating mast cells through chemotaxis, stimulating cytokine and antibody production, enhancing keratinocyte survival to prevent cell death, and neutralizing bacterial lipopolysaccharides to mitigate inflammation (Figure 5).

Another notable AMP is hepcidin, synthesized by MSCs and essential for iron homeostasis. Hepcidin reduces intestinal iron absorption and mobilizes stored iron, restricting bacterial access to this vital nutrient during infections [100]. The development of resistance-free AMPs is a critical area of research. Techniques such as functional metagenomics, systematic gene overexpression, and laboratory evolution have been employed to engineer AMPs with reduced susceptibility to bacterial resistance. These modified AMPs are characterized by lower polar and positively charged amino acids and increased hydrophobicity, which enhance their antimicrobial activity while minimizing resistance development [100]. The combination of MSCs and AMPs offers a synergistic approach to combating AMR. MSCs not only produce AMPs like β-defensin, LL-37, and hepcidin, but also act as delivery systems that enhance AMP efficacy in targeted treatments. This dual-action approach has shown promise in treating chronic infections and biofilm-associated diseases.

### 4.4. Immunotherapeutic Approaches

Even bacteria that are susceptible to antibiotics can evade treatment by employing mechanisms to bypass the immune system. To address this challenge, immunotherapeutic approaches focus on leveraging and enhancing the immune system’s ability to combat bacterial infections. Adaptive lymphocytes, such as T and B cells, and innate immune cells, including innate lymphoid cells (ILCs) and natural killer (NK) cells, play a central role in regulating and eliminating infections. They achieve this by directly killing infected cells and secreting inflammatory molecules that stimulate or reinitiate bactericidal responses from myeloid cells. Importantly, a balance must be maintained to prevent excessive inflammation, which can cause tissue damage. This balance is mediated by pathways such as PD-1/PD-L1, which help to modulate immune activity. For instance, the upregulation of PD-1 in T cells during Mycobacterium tuberculosis infections is essential to avoid immune-mediated tissue damage [101].

Recent studies have explored the immunomodulatory potential of the Tim3-Gal9 axis in antimicrobial immunity. Tim3, a T cell immunoglobulin and mucin domain protein, serves as a negative regulator that suppresses effector TH1-type immune responses. While this suppression helps mitigate inflammation-related tissue damage, the overactivation of Tim3 can lead to T cell exhaustion. Galectin-9 (Gal9), a ligand of Tim3, interacts with Tim3 on TH1 cells, triggering macrophages to enhance their bactericidal activity. This process is mediated through the caspase–1-dependent secretion of IL-1β, a cytokine critical for antimicrobial responses. The coupling of Tim3 and Gal9 has been shown to increase antimicrobial immunity by both stimulating macrophages and balancing immune responses [102]. Another innovative method combines antibacterial sonodynamic therapy with antivirulence immunotherapy by using engineered nanovesicles to capture bacterial toxins and generate reactive oxygen species upon ultrasound activation [103].

Macrophages, a key component of the innate immune system, exhibit remarkable plasticity and can polarize into two distinct phenotypes in response to bacterial infections. The M1 phenotype, or classically activated macrophages, promotes pro-inflammatory responses, producing cytokines that attack pathogens and activate other immune cells. In contrast, the M2 phenotype, or alternatively activated macrophages, is associated with anti-inflammatory activities, tissue repair, and the resolution of inflammation. The therapeutic modulation of macrophage polarization is an emerging strategy in immunotherapy. Encouraging the M1 phenotype is crucial for effective pathogen clearance during the early stages of infection, while shifting macrophage activation toward the M2 phenotype can help resolve inflammation and repair tissue damage [104].

### 4.5. Antibacterial Photodynamic Therapy

**Figure 6:**
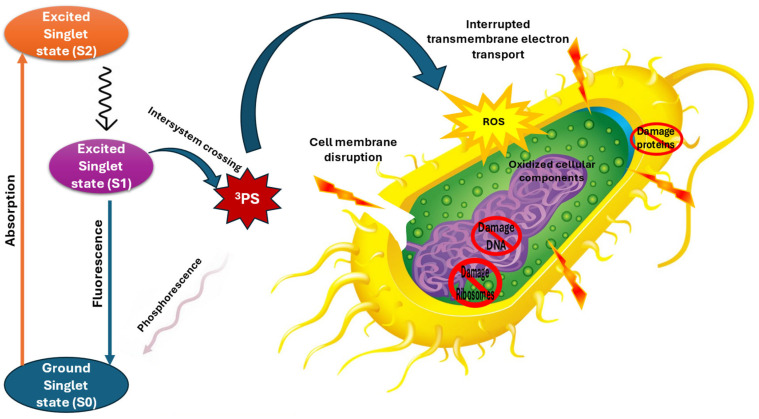
Schematic illustration of antimicrobial photodynamic therapy.

Photodynamic therapy (PDT) emerged serendipitously in the early 1900s, revealing the profound potential of light-activated chemicals to induce cellular destruction [105]. This innovative therapeutic approach represents a sophisticated intersection of photochemistry, molecular biology, and targeted treatment strategies. The fundamental efficacy of PDT hinges on the intricate interaction between three critical components: a non-toxic photosensitizer (PS), specific wavelength light, and molecular oxygen within biological systems. The mechanism of PDT is a complex photochemical process that begins when a photosensitizer is exposed to light of a particular wavelength. Upon light activation, the photosensitizer transitions into an excited triplet state, initiating a cascade of molecular interactions. This exciting state becomes a powerful catalyst, transferring energy to surrounding oxygen molecules and generating highly reactive oxygen species (ROS). These unstable molecular entities, including singlet oxygen and hydroxyl radicals, possess remarkable destructive capabilities, which are capable of oxidizing and damaging critical biomolecules, cellular structures, and even drug-resistant pathogens (Figure 6) (Table 3). The generation of ROS introduces additional chemical dynamics, particularly in the presence of iodide anions. These interactions can produce unstable iodide radicals, forming triiodide and hydrogen peroxide, which further contribute to cellular damage mechanisms. However, the therapeutic potential of PDT is intricately linked to tissue oxygen concentrations, presenting both opportunities and challenges in clinical applications [106].

While PDT demonstrates significant promise, certain limitations currently constrain its widespread clinical implementation. Long absorption latency and extended exposure times can potentially diminish its practical utility. The therapy’s effectiveness is fundamentally determined by three interdependent factors: photosensitizer absorption characteristics, light energy properties, and intracellular oxygen content. Recent research has explored innovative strategies to enhance PDT’s antimicrobial potential, particularly in combating resistant bacterial strains. A compelling approach involves combining PDT with traditional antibiotics, creating synergistic therapeutic protocols. For instance, studies examining the combination of toluidine blue (TB)-based antibacterial photodynamic therapy (aPDT) with gentamicin (GEN) have yielded promising results.

In vitro and in vivo investigations demonstrated that the combined GEN and aPDT treatment could effectively inhibit the growth of both standard and multidrug-resistant *Staphylococcus aureus* for up to 15 h. This combined approach demonstrated remarkable capabilities in destroying bacterial cell envelopes and disrupting biofilm structures, highlighting the potential of integrated therapeutic strategies. The underlying mechanism of this combined approach relies heavily on ROS-induced oxidative stress. By generating reactive oxygen species through non-toxic photosensitizers and visible light, aPDT can fatally damage bacterial cells. Gentamicin, a traditional aminoglycoside antibiotic, complements this approach by inhibiting microbial ribosomal function, thereby accelerating pathogen elimination [107]. Notably, the oxidative stress generated by aPDT can attack critical bacterial envelope proteins and lipids, compromising membrane transport systems and cellular integrity. This multifaceted assault significantly reduces bacterial virulence and infectivity. The potential clinical implications are substantial, suggesting that such combined therapies could potentially reduce antibiotic dosages required for infection clearance while simultaneously promoting more efficient wound healing [107]. The oxidative mechanisms of PDT extend beyond immediate bacterial destruction. By reducing bacterial colonization, modulating inflammatory factors, and stimulating growth factor expression, these therapies represent a sophisticated approach to managing complex infectious challenges.

As research continues to evolve, photodynamic therapy stands at the forefront of innovative antimicrobial strategies, offering hope in an era increasingly challenged by antibiotic-resistant pathogens. The continued exploration of PDT’s mechanisms, refinement of photosensitizer technologies, and development of targeted therapeutic protocols promise exciting advancements in medical treatment paradigms.

**Table 3:** Photodynamic therapy: a comprehensive overview of photosensitizers, bacterial targets, and light sources.

Photosensitizer	Pathogenic Bacteria	Light Type	Wavelength (NM)	Key Findings	Reference
Gentamicin	*Staphylococcus aureus*	Blue Light	630–660	Effective against standard and MDR strains	[108]
Methylene blue	*Escherichia coli*	Red Light	660–670	Significant membrane damage	[109]
Chlorin e6	Methicillin-Resistant *S. aureus*	Red Light	665–685	Enhanced ROS generation	[110]
Phthalocyanine	Vancomycin-Resistant Enterococci	Far-Red Light	670–690	Effective against resistant strains	[111]
5-aminolevulinic acid	Carbapenem-Resistant *Acinetobacter baumannii*	Red Light	630–635	Significant bacterial reduction	[112]
Aluminum phthalocyanine chloride	Multidrug-Resistant *Enterobacter*	Near-Infrared	670–690	Improved tissue penetration	[113]

### 4.6. Relationship Between CRISPR CAS (Adaptive Immunity of Bacteria) and Antibiotic Resistance

**Figure 7:**
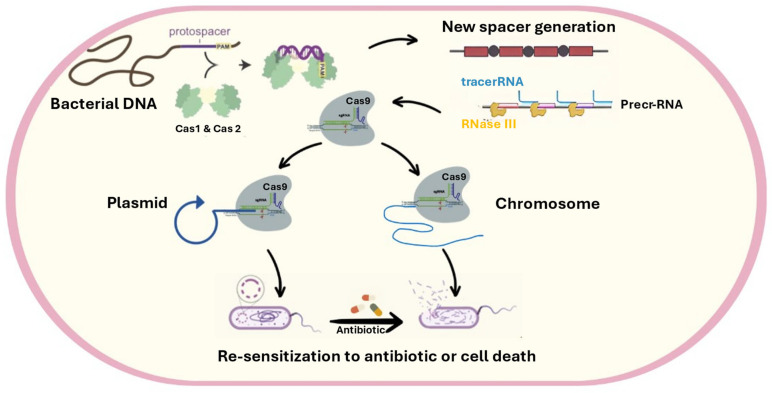
CRISPR-Cas systems as antimicrobial agents. CRISPR-Cas targets bacterial resistance genes by integrating foreign DNA sequences into the CRISPR array. Processed crRNA guides Cas9 to cleave matching DNA in plasmids or chromosomes, disrupting resistance genes. This leads to re-sensitization to antibiotics or bacterial cell death, offering a targeted strategy against antibiotic resistance.

CRISPR-Cas (clustered regularly interspaced short palindromic repeats and their associated Cas proteins) are an adaptive immune system found in bacteria that helps protect them from invasive genetic material such as bacteriophages and plasmids [114]. It acts as a natural barrier to horizontal gene transfer, a common mechanism for spreading antibiotic resistance [115]. This suggests that CRISPR-Cas may play a role in limiting the acquisition and dissemination of antibiotic resistance genes among bacterial populations. Moreover, unlike traditional antibiotics, which often lack specificity and harm beneficial bacteria, CRISPR-Cas systems can directly and selectively target antibiotic resistance genes (*ARGs*) and eliminate pathogenic bacteria without affecting other bacterial species in complex bacterial populations. This is because CRISPR-Cas systems are guided by RNA molecules complementary to specific DNA sequences, allowing for the precise targeting of ARGs (Figure 7) [116].

The specificity of CRISPR-Cas systems makes them a promising tool for combating antibiotic resistance. By targeting and eliminating ARGs, CRISPR-Cas can restore the effectiveness of traditional antibiotics and improve the treatment of bacterial infections. CRISPR-Cas systems can be engineered to target multiple ARGs simultaneously, making them effective against bacteria that have developed resistance to multiple antibiotics [117].

Research is ongoing to develop CRISPR-Cas systems for clinical use. These systems have the potential to revolutionize the treatment of antibiotic-resistant infections and improve global health outcomes.

One of the main factors contributing to the growth of multidrug resistance among bacterial pathogens is the acquisition of antibiotic resistance (*ABR*) genes through horizontal gene transfer (*HGT*). CRISPR-Cas, as an adaptive immune system, comprises CRISPR loci and CRISPR-associated (*cas*) genes present in around 30–40% of bacteria [118] between repeating sequences loci. Serving as a kind of “memory” for the immune system, any sequence that matches this immunological record is cut and destroyed by the spacer sequences’ cognate Cas enzymes. By doing so, the cell can protect itself from bacteriophage attack and prevent the entry of additional and possibly expensive Mobile Genetic Elements (*MGEs*) [118].

*Acinetobacter baumannii* utilizes the I-Fb CRISPR-Cas system to impede the spread of antibiotic resistance genes. This CRISPR-Cas system effectively halts the horizontal transfer of genetic elements and is highly conserved. The removal of any component of the CRISPR-Cas system resulted in the decreased outer membrane permeability of AB43 strains, aligning with previous findings on membrane permeability [119].

Recent research has demonstrated a correlation between the presence of the CRISPR-Cas system and a reduction in antibiotic resistance genes, along with a significant decrease in virulence factors. Conversely, the absence of the CRISPR locus was found to be linked to multidrug resistance. Furthermore, an inverse relationship exists between the prevalence of bacterial resistance and the existence of the CRISPR-Cas system [115].

Additionally, targeting the quorum-sensing regulator *lasR* mRNA has shown promise in reducing bacterial pathogenicity in *Pseudomonas aeruginosa*, particularly when utilizing the I-F CRISPR-Cas system. Interestingly, the *QS* synthase gene abaI contains a region that partially matches with spacer 101 of the CRISPR system, specifically, nucleotides 29 to 39 in abaI and its repeats. This suggests a potential mechanism for the CRISPR-mediated regulation of quorum sensing and pathogenicity in *P. aeruginosa.* Targeting the quorum-sensing regulator lasR mRNA has been shown to reduce bacterial pathogenicity in Pseudomonas aeruginosa, particularly when utilizing the I-F CRISPR-Cas system. Interestingly, the *QS* synthase gene abaI contains a region that partially matches with spacer 101 of the CRISPR system, specifically, nucleotides 29 to 39 in abaI and its repeats. Eliminating any component of the CRISPR-Cas system renders the strain highly resistant to the majority of the medicines tested. The I-Fb CRISPR-Cas system may also target and degrade abaI (*QS* synthase) mRNA, thereby reducing biological features and genes associated with drug resistance due to the decreased level of *AHLs*. Furthermore, Cas3 cleavage activity was found to be crucial in controlling the degradation of abaI mRNA [119].

The number of CRISPR loci was found to be significantly higher in gentamicin-, teicoplanin-, erythromycin-, and tetracycline-susceptible strains. Conversely, a smaller number of CRISPR loci were identified in strains positive for vancomycin (*vanA*), tetracycline (*tetM*), macrolides *(ermB*), aminoglycosides (*aac6’-aph(2”)*), streptogramins (*aadE*), and streptothricin (*ant(6)*), indicating a negative correlation between CRISPR-Cas loci and antibiotic resistance [120].

The majority of the *Staphylococcus coagulans* isolates that were methicillin-susceptible had Type IIC CRISPR-Cas systems and no genes for antibiotic resistance. This suggests that the Type IIC CRISPR–Cas system in *S. coagulans* strains may hinder the acquisition of antibiotic-resistant genes (*ARG*) containing mobile genomic elements [121].

Furthermore, *Enterococci isolates* exhibited CRISPR-Cas components in 63.5% (54/85) of cases. The study revealed a significant inverse relationship between the presence of CRISPR-Cas elements and the proportion of *E. faecalis* isolates resistant to antibiotics such as vancomycin, ampicillin, chloramphenicol, erythromycin, rifampin, teicoplanin, tetracycline, imipenem, tigecycline, and trimethoprim-sulfamethoxazole. Moreover, compared to CRISPR-Cas negative E. faecium strains, CRISPR-Cas positive *E. faecium* isolates exhibited significantly lower rates of resistance to vancomycin, ampicillin, chloramphenicol, erythromycin, teicoplanin, and tetracycline [122].

In *E. faecalis*, the consensus repeats sequences of the CRISPR1 and CRISPR2 loci are identical, suggesting a functional connection between the two loci. An orphan CRISPR2 locus alone cannot effectively defend the genome against mobile genomic elements; it requires the support of CRISPR1-cas. Interestingly, CRISPR3, rather than CRISPR1, was associated with the absence of antibiotic resistance. The availability of the CRISPR3-mutant T11, which acquired cas9 (*cas9* + *CRISPR3*) for interference, indicates that CRISPR3-cas is active for sequence-specific genome defense. Deleting just two loci can significantly weaken the genome’s ability to protect itself from clinically mobile genomic fragments [120].

Figure 7 illustrates how CRISPR-Cas systems can potentially function as antibiotics. The *Cas9* enzyme, which can cut DNA in a specific location guided by an RNA molecule, is expressed along with a guide RNA designed to target a particular DNA sequence. The targeted sequence could be on a plasmid or the main bacterial chromosome. The cutting of the plasmid or chromosome by *Cas9* can lead to the degradation of the genetic material, resulting in cell death or loss of antibiotic resistance encoded by genes on the targeted DNA. Therefore, the CRISPR-Cas9 system can selectively degrade DNA in bacterial cells, providing an antibacterial mechanism.

### 4.7. Bacteriophages and Their Role in Antibiotic Resistance and Sensitivity

Bacteriophages, or phages, are viruses that specifically infect and replicate within bacterial cells. These natural predators of bacteria have garnered significant attention for their potential role in combating antibiotic-resistant infections. Phage therapy and antibiotic resistance phage therapy, the use of bacteriophages to treat bacterial infections, have emerged as a promising alternative to traditional antibiotics, particularly in the face of growing antimicrobial resistance [123]. Phages can selectively target and kill specific bacterial strains, including those resistant to multiple antibiotics. This specificity helps preserve the host’s beneficial gut microbiome, unlike broad-spectrum antibiotics that can disrupt the natural microbial balance [124]. Several studies have highlighted the ability of phages to overcome antibiotic resistance. For example, a review by Dufour et al. [125] discussed the successful use of phage therapy to treat infections caused by multidrug-resistant *Pseudomonas aeruginosa*, *Acinetobacter baumannii*, and *Klebsiella pneumoniae*. The authors noted that phages can employ various mechanisms to combat resistance, such as directly targeting resistant determinants or disrupting resistance pathways. The combination of phages and antibiotics, known as phage-antibiotic synergy (PAS), has emerged as a particularly effective strategy against antibiotic-resistant bacteria [124]. By using phages and antibiotics concurrently, researchers have observed enhanced antimicrobial activity and the ability to overcome resistance mechanisms.

A study by Oechslin et al. [126] demonstrated the potential of PAS in treating acute pneumonia caused by multidrug-resistant *P. aeruginosa* in a mouse model. The combination of a phage cocktail and the antibiotic ciprofloxacin resulted in a significant reduction in bacterial load and improved survival compared to either treatment alone. The synergistic effects of phages and antibiotics can be attributed to several mechanisms. Phages can sensitize bacteria to antibiotics by disrupting cell membranes, altering gene expression, or inducing the formation of persisted cells that are more susceptible to antibiotics [127]. Conversely, antibiotics can increase phage adsorption to bacterial cells or enhance phage replication, leading to a more effective antimicrobial response [124]. While phages offer promising solutions for combating antibiotic resistance, it is important to consider their potential role in the spread of resistance genes. Phages can act as vectors for horizontal gene transfer, allowing for the dissemination of resistance determinants across bacterial populations [128]. Bacteriophages can acquire resistance genes from their bacterial hosts and, subsequently, transfer these genes to other bacteria through transduction, a process in which phages package and deliver bacterial genetic material [129]. This exchange of genetic material can contribute to the emergence and spread of antibiotic-resistant strains, underscoring the need for careful consideration and monitoring when employing phage therapy.

Bacteriophages have emerged as a promising alternative to traditional antibiotics, with the potential to overcome antibiotic resistance through various mechanisms. The synergistic use of phages and antibiotics has shown particular promise in combating multidrug-resistant infections. However, the role of phages in the dissemination of resistance genes highlights the importance of a balanced and well-informed approach to phage therapy. Continued research and the careful implementation of phage-based interventions will be crucial in harnessing the full potential of these natural bacterial predators in the fight against antimicrobial resistance.

### 4.8. Animal Venoms

Although animal venom is extremely costly and deadly, it offers many interesting possibilities for the creation of biotherapeutics. The “peptides”, which have demonstrated a wide range of biological activities, potential site particularity, and involvement in regulating biological mechanisms, are one of the main ingredients of scorpion venom. In particular, these peptides have drawn increased interest in developing antibiotic-resistant solutions [130]. Animal venoms, which have been utilized for decades in traditional medicine to treat infections and viruses, are one example of the many bioactive substances found in nature [131]. Antimicrobial peptides (AMP) are a promising option for novel therapeutic alternatives in these kinds of instances. AMPs, which are antimicrobial agents with an average length of less than 100 amino acids, are derived from the venoms of several animals, including scorpions, bees, spiders, and snakes [132]. AMPs are short cationic amphipathic peptides found in venoms. Based on their structure, they are categorized into three groups: (1) peptides with cysteine residues and disulfide bridges; (2) peptides without cysteine residues that include members with amphipathic α-helix; and (3) peptides with proline and glycine residues that are abundant in the second group. Three to four disulfide bridges combine to generate cysteine-containing AMPs [133].

#### 4.8.1. Scorpion Venom

Scorpion venom has shown promise in combating antimicrobial resistance (AMR) in bacteria. The venom contains peptides known as α-toxins and β-toxins that disrupt vital bacterial processes, making them effective against multidrug-resistant (MDR) pathogens.

α-Toxins, or sodium channel toxins, can modulate the gating properties of voltage-gated sodium channels in bacterial cell membranes, leading to membrane depolarization and the disruption of essential cellular processes. Several studies have demonstrated the antimicrobial activity of α-toxins against MDR bacteria, including methicillin-resistant Staphylococcus aureus (MRSA) and extended-spectrum beta-lactamase (ESBL)-producing Gram-negative bacteria [134].

Similarly, β-toxins, or potassium channel toxins, target bacterial potassium channels, interfering with membrane potential regulation and metabolic pathways. Recent research has shown the efficacy of β-toxins against MDR bacteria, such as *Acinetobacter baumannii*, *Klebsiella pneumoniae*, and *Pseudomonas aeruginosa* [134]. Interestingly, the combination of α-toxins and β-toxins can produce synergistic effects, enhancing their antimicrobial potency against MDR bacteria by simultaneously disrupting multiple cellular processes [134].

Furthermore, scorpion venom peptides have demonstrated insecticidal activities, with Meucin-49 from *Mesobuthus eupeus* exhibiting broad-spectrum action against both Gram-positive and Gram-negative bacteria, as well as activity against the bacterial symbionts of pea aphids (*Acyrthosiphon pisum*), a serious agricultural pest [135]. Additionally, peptides from the venom of the Australian scorpion *Urodacus yaschenkoi*, such as UyCT1, UyCT3, and UyCT5, have been shown to effectively inhibit Acinetobacter baumannii and other MDR infections [136]. The BmKn-22 peptide, a modified version of the parental BmKn-2 scorpion venom peptide, has also demonstrated the ability to inhibit the development of and destroy pre-formed *Pseudomonas aeruginosa* biofilms [137]. Overall, the unique mechanisms of action and the potential for synergistic effects make scorpion-venom-derived compounds a promising avenue for combating antimicrobial resistance.

#### 4.8.2. Bee Venom

One of the many bee products high in bioactive compounds is bee venom, a naturally occurring material with a range of actions against various disease etiology [138]. Bee venom is a complex mixture of bioactive compounds, including peptides, enzymes, and other small molecules. Among these components, melittin and apamin peptides have gained significant attention due to their potential antimicrobial properties and ability to modulate immune responses [139] (Table 4). Bv and its main constituents, PLA2 and melittin, were used to combat oral infections found to be the root causes of tooth decay. Between 20 and 40 µg/mL is the minimum inhibitory concentration (MIC) of the BV against *Lactobacillus casei*, *Enterococcus faecalis*, *Streptococcus mitis*, *Streptococcus sanguinis*, *Streotococcus sobrinus*, and *Streptococcus salivarius* [140].

Bee venom has been shown to possess significant antibacterial activity against a range of multidrug-resistant bacteria. Hegazi et al. also demonstrated the antibacterial activity of bee venom against various pathogenic bacterial strains [141]. Fadl further confirmed this, showing that bee venom has a strong potential effect against MDR isolates, including Gram-negative and Gram-positive bacteria. Another study highlighted the potential of melittin, a component of bee venom, as an antimicrobial agent, particularly in treating MRSA infections [142]. These studies collectively suggest that bee venom and its components could be valuable in the fight against multidrug-resistant bacteria [143].

#### 4.8.3. Snake Venom

Snake venom contains a variety of antimicrobial proteins and peptides, including defensins and cathelicidins, which have shown potential for developing alternative antimicrobial agents. Defensins are small cationic peptides active against a broad spectrum of bacteria, including Gram-positive and Gram-negative bacteria. They are typically 30–45 amino acids in length and have a conserved cysteine-rich motif [144]. Cathelicidins are another type of antimicrobial peptide produced by various organisms, including snakes. They are typically larger than defensins, ranging from 12 to 80 amino acids in length, and have a more variable structure, but contain a conserved cathelin domain [145].

These antimicrobial peptides have been observed in various snake species, including the common night adder, gaboon adder, and black mamba, among others [146]. One important class of AMPs found in snake venoms is cathelicidins, such as cathelicidin-NA from the Chinese cobra and cathelicidin-BF from the Bungarus fasciatus snake, which exhibit broad-spectrum antimicrobial activity against bacteria, fungi, and viruses [132].

Additionally, crotamines, cationic peptides isolated from the venom of the South American rattlesnake *Crotalus durissus* terrificus, have demonstrated potent antimicrobial activity against various bacterial strains, including multidrug-resistant pathogens [147]. Crotamines have also been found to inhibit the development of *Trypanosoma cruzi*, the causative agent of Chagas disease [148]. Other components of snake venom, such as phospholipase A2 (PLA2) enzymes and L-amino acid oxidases (LAAOs), have also exhibited antimicrobial potential. PLA2s can disrupt bacterial cell membranes, while LAAOs can generate oxidative stress and damage bacterial cells [149,150].

Furthermore, snake venom metalloproteases, like zinc-dependent metalloproteinases (SVMPs), have shown activity against Gram-positive and Gram-negative bacteria, including multidrug-resistant strains, by disrupting cell membranes and degrading essential proteins [151]. Overall, the diverse array of antimicrobial compounds found in snake venom represents a promising avenue for the development of novel therapeutic agents to combat antimicrobial resistance.

#### 4.8.4. Spider Venom

Spiders are known to produce a diverse array of venom peptides, including a family of cationic peptides called insect-selective cystine-knot (ICK) peptides. These peptides have garnered significant interest due to their potential as antimicrobial agents, particularly against multidrug-resistant (MDR) bacteria (Table 4). ICK peptides are characterized by their unique knotted structure, formed by the interlocking of disulfide bridges. This structural motif confers remarkable stability and resistance to proteolytic degradation, making them attractive candidates for therapeutic applications [132]. Several studies have demonstrated the antimicrobial activity of ICK peptides against a range of MDR bacteria, including Gram-positive and Gram-negative pathogens. Here are some examples of ICK peptides from spiders and their potential as antimicrobial agents. Wang et al. [152] isolated and characterized lycosin-II from the venom of the spider *Lycosa singoriensis*, which displayed potent bacteriostatic effects on drug-resistant bacterial strains. Similarly, Abreu et al. [153] identified gomesin with potential antimicrobial activity in the venom of the Brazilian spider *Acanthoscurria gomesiana*. These findings suggest that spider venom peptides have the potential to be developed into novel antibiotics. Wu et al. [154] further support this, noting the diverse pharmacological activities of spider venom peptides, including their potential as anticancer and antinociceptive agents.

**Table 4:** Selected AMPs from animal venom showing their antimicrobial activity.

Venom Species	Peptides’ Name	Amino Acid Residues	Antimicrobial Activity	MIC	Reference
Scorpion (*Hoffmannihadrurus aztecus*)	Hadurin	41 amino acid-long AMP	Antimicrobial activity was mainly detected against *Escherichia coli*, *Serratia marscencens*, and *Enterococcus cloacae*	Lower than 10 µm	[155]
Scorpion (*Pandinus imperator*)	Pandinin-1	44 amino acids	Antimicrobial activity was mainly detected against *Enterococcus faecalis*, *Bacillus subtilis*, *Staphylococcus aureus*, and *Staphylococcus epidermidis*	1.3 µm, 5.2 µm, 2.6 µm, and 5.2 µm, respectively, according to those species	[156]
Scorpion *(Pandinus imperator*)	Pandinin-2,	24 amino acid residues	Antimicrobial activity was mainly detected against *Enterococcus faecalis*, *Bacillus subtilis*, *Staphylococcus aureus*, and *Staphylococcus epidermidis* strains and *Mycobacterium tuberculosis*	2.4 µm, 4.8 µm, 2.4 µm and 4.8 µm, respectively, according to those species	[157]
Scorpion (*Mesobuthus martensii)*	Bmkbpp	47 amino acid residues	Antimicrobial activity was mainly detected against Gram-negative bacteria	2.3 to 68.2 µm for different strains	[158]
Scorpion (*Vaejovis punctatus)*	Vpamp1.0 and vpamp2.0	19 to 25 amino acid residues	Antimicrobial activity was mainly detected against Gram-positive and Gram-negative bacteria	2.5 to 15 µm and 2.5 to 24 µm	[159]
Scorpion (*Tityus serrulatus)*	Tsap-2	17 amino acid residues	Antimicrobial activity was mainly detected against *Staphylococcus aureus*	5 µm	[160]
Scorpion (*Scorpiops tibetanus)*	CT2	14 amino acid residues	Inhibits mainly Gram-positive bacteria, especially *Staphylococcus aureus* Effective against methicillin-resistant bacterial strains	6.25 μg/mL	[161]
Bee *(Apis mellifera)*	Melittin	26 amino acids	Broad-spectrum antimicrobial activity against Gram-positive and Gram-negative bacteria, as well as fungi	0.5–100 μg/mL against various bacteria	[162]
Bee *(Apis mellifera)*	Apidaecin	18 amino acids	Potent activity against Gram-negative bacteria, particularly Escherichia coli and Salmonella spp.	1.25–5 μg/mL against *E. coli*	[163]
Snake *(Bothrops atrox)*	Cathelicidin-NA	34 amino acids	Antimicrobial activity against Gram-positive and Gram-negative bacteria, as well as fungi	0.25–128 µg/mL	[164]
Snake *(Naja atra*)	Cathelicidin-NA	34 amino acids	*Bacillus anthracis*	0.29 µg/mL	[165]
Snake *(Ophiophagus hannah*)	Cathelicidin-NA	-	*P. Aeruginosa*	3.25 µm	[166]
Snake *(Crotalus durissus terrificus)*	Crotamine	42 amino acids	*Citrobacter freundii*, *B. Subtilis*, and *Micrococcus luteus*	-	[144]
Spider *(Acanthoscurria paulensis)*	Gomesin	18 amino acids	Potent antimicrobial activity against Gram-positive and Gram-negative bacteria, as well as fungi	0.8–6.4 μm against various bacteria	[153]
Spider *(Cupiennius salei)*	Cupiennin 1a	35 amino acids	Antimicrobial activity against Gram-positive and Gram-negative bacteria, as well as fungi	0.5–4 μm against various bacteria	[167]
Spider *(Psalmopoeus cambridgei)*	Psalmopeotoxin I	28 amino acids	Antimicrobial activity against Gram-positive and Gram-negative bacteria, as well as fungi	0.4–12.5 μm against various bacteria	[168]

In the future, a promising strategy to mitigate the cytotoxic effects of traditional antibiotics on eukaryotic cells involves combining low quantities of fast-killing scorpion antimicrobial peptides (AMPs) with traditional antibiotics. This innovative approach aims to address the challenge of multidrug-resistant (MDR) infections by leveraging the unique properties of scorpion-derived peptides. By utilizing AMPs from animal venom, which are known for their potent antimicrobial activity against a wide range of pathogens, alongside conventional antibiotics, it may be possible to achieve synergistic effects that enhance the efficacy of treatment while minimizing adverse effects on host cells.

#### 4.8.5. Potential Side Effects of Venom-Based Antimicrobial Agents

While venom-derived compounds show promise in combating antibiotic resistance, their potential side effects on humans must be considered. Many venom peptides exhibit cytotoxicity toward mammalian cells, which can lead to hemolysis, neurotoxicity, or immunogenic responses. Bee venom, for instance, has shown promising antimicrobial effects, but may cause allergic reactions and cytotoxicity in high doses [140]. Similarly, lionfish venom contains bioactive peptides with antimicrobial properties, but its toxic side effects, including hemolysis and pain, remain concerns [169]. Additionally, spider venom peptides demonstrate antibacterial and anti-inflammatory effects, but require further investigation into their safety profiles [170]. Therefore, while venoms hold the potential for combating antibiotic resistance, their adverse effects must be carefully evaluated for clinical applications.

To mitigate toxicity risks, researchers are employing peptide modifications to enhance selectivity toward bacterial membranes while minimizing harm to human cells. Chemical modifications, such as amino acid substitutions and structural optimization, have been shown to improve antimicrobial activity while reducing cytotoxicity [171]. Additionally, nanocarrier-based delivery systems, including self-assembled peptide nanostructures, have been developed to control drug release and minimize off-target effects [172]. These approaches collectively improve the clinical viability of antimicrobial peptides while addressing safety concerns. Additionally, the successful development of venom-derived drugs like Captopril and Ziconotide demonstrates that, with rigorous clinical testing, venoms can be safely harnessed for therapeutic applications [151]. Further studies are essential to evaluate the clinical safety profile of venom-based antibiotics before their therapeutic application.

### 4.9. Nanobiotics

Nanoparticles (NPs) have emerged as a promising platform for the controlled delivery of antibiotics and other therapeutic agents due to their unique properties and versatility. NPs possess several advantages, including their ability to encapsulate and protect drugs from degradation, enhance solubility and bioavailability, and target specific sites or cells within the body. Additionally, some NPs exhibit inherent antimicrobial properties, making them valuable tools in combating bacterial infections. Nanobiotics offer a promising approach to combat antimicrobial resistance (AMR) by providing several advantages over conventional antibiotics, which are summarized in Table 5.

There are four main categories of NPs: inorganic (metallic NPs, silica NPs, and Mesoporous silica NPs), organic (polymeric NPs, liposomes, and dendrimers), carbon-based (carbon nanotubes, Graphene NPs, and Fullerenes), and composite (metal, polymer, and ceramic) [177]. The efficacy of these NPs in drug delivery and antimicrobial applications depends on various factors, including their size, shape, surface chemistry, drug loading capacity, release kinetics, and targeting specificity. Additionally, the inherent antimicrobial properties of certain NPs, such as silver and copper, can be exploited to enhance their therapeutic potential against bacterial infections. Nanobiotics lead to an increase in the medications’ bioavailability in the intended tissue, preventing drug deterioration and reducing the harmful effects on patients. Antibiotics can increase their antibacterial effectiveness by loading them into nanocarriers, according to several studies. When delivering novel antibacterial modalities to bacteria, “nanobiotics” refer to the use of intentionally generated pure antibiotics with a size range of ≤100 nm or antibiotic molecules enclosed with man-made nanoparticles (NPs). These techniques present special chances to fight infections caused by planktonic and multidrug-resistant biofilms [178].

The chemical composition of antibiotic-loaded nanocarriers may raise concerns about immunological action, cytotoxicity, biocompatibility, and biodegradability, even if they may minimize the dosage needed to eliminate bacterial resistance [178].

Compared to conventional antibiotics, antimicrobial nanoparticles (NPs) provide numerous exceptional advantages in overcoming resistance and reducing expenses [179]. In order to improve the pharmacokinetic characteristics of antibiotics and reduce their adverse effects, a variety of nanosized drug carriers are currently available [180]. The utilization of antibacterial nanoscale materials holds significant potential in addressing life-threatening infections. These materials are strategically engineered to enhance biofilm penetration and disrupt bacterial systems effectively. To safely manage superficial infections and infectious disorders, the preference lies in employing metals at the nanoscale, with metal oxides, organic nanoparticles, and nanocomposites all possessing robust antibacterial properties. The diverse chemical compositions and inherent characteristics of these antibacterial nanomaterials, commonly referred to as nanobiotics, offer versatile strategies for combating targeted bacteria [181]. When compared to using antibiotics in bulk, “antibiotic nanocarriers” based on liposomal, solid/lipid, terpenoid, polymeric, dendrimeric, and inorganic materials have demonstrated positive results in improving the total efficacy of antibiotics [182]. NPs can either actively or passively target disease areas. Because infected locations have higher nanocarrier permeability than uninfected tissue, on-target accumulation results are improved. It is also possible to functionalize ligands (such as antibodies) on nanocarrier surfaces that bind to sick tissues or microbes as receptors (such as antigens). Active targeting or ligand-mediated targeting refers to the latter strategy.

Additionally, ligand-conjugated nanocarriers have the potential to enhance intracellular infection treatment by improving cell absorption. Targeted drug delivery systems have advanced quickly in recent years, primarily in the treatment of cancer and tuberculosis caused by Mycobacterium tuberculosis [174,183]. The conjugation was more effective than the antibiotic in its free form. Most importantly, the targeted therapy seems to affect intracellular bacteria that the antibiotic would have missed and left “hidden,” acting as a latent cause of recurrent sickness [184].

Because inorganic NPs are composed of the inorganic oxides of Ag, Mn, Al, Ti, Se, Au, Si, or Cu, their size, shape, solubility, and stability vary. Their antibacterial efficacy is also influenced by aggregation behavior, pH, temperature, reduction time, and reducing agent concentration, all of which determine their properties. To impede respiration, including bacterial cell lysis and inducing inflammatory responses, AgNPs, for instance, adhere to cell membranes, interact with membrane proteins, raise membrane porosity, and enter and promote ROS formation [184]. Since ancient times, silver has demonstrated immense promise for use as an antibacterial and antiseptic agent. Because of the antibacterial effect of bacterial AgNPs, there may be a special way to minimize the development of antibiotic resistance by using fewer drugs overall [185]. AgNPs act as a delivery system for ampicillin (amp) antibiotic with a spherical shape and 4 nm and coated with citrate that can load 1.06 × 10^−6^ of the amp to target *Pseudomonas aeruginosa*, *Escherichia coli*, and *Vibrio cholerae* to target their cell walls and β-Lactamase species [186].

Because of strong electrostatic forces that cause intracellular loss and cell death, gold nanoparticles (AuNPs) are known to have bactericidal effects [187]. AuNPs accumulate on the surface of cells and have bactericidal effects. The antibacterial properties of Au nanocrystals are facet-dependent and include bacterial membrane damage, the suppression of cellular enzyme activity, and energy metabolism [188]. AuNPs with 4–5 nm and spherical size represent an example of delivering the vancomycin (Van) antibiotic. The phenyl group of Van attaches to AuNP via the Au-S bond, and each AuNP links with approximately 31 Van on its surface to target *Escherichia coli* and Vancomycin-resistant Enterococci, and targets their cell membranes [189].

For instance, iron oxide NPs (FeONPs) and DNA hybridization are combined to increase the bacterial 16S ribosomal RNA gene capture [190] due to strong electrostatic forces, cytoplasmic leakage, cell death, and gold nanoparticles. Because CaF2 NPs stick to tooth surfaces and release fluoride ions continuously, they have been demonstrated to have fatal effects on *Streptococcus mutans*. This promotes remineralization and inhibits pathogenic *S. mutans* [191]. Because of their hydrophilic/hydrophobic individualities, most organic NPs, including liposomes, polymeric materials, and micelles, are biocompatible and rapidly opsonize [181].

Liposomes and lipid nanoparticles are phospholipid bilayers in spherical vesicle form. The medication can be released into bacteria when they fuse with the microbial membrane. They have been transformed into a medication delivery system to combat illnesses caused by biofilms using antimicrobial medicines. By reducing recurrent infections, their special qualities—such as target specificity, low toxicity, and the capacity to fuse biofilm matrix/cell membrane—improve the effectiveness of antibiotics [192].

Drug carriers are polymeric nanoparticles (NPs), such as nanospheres and nanocapsules. They improve the effectiveness of targeting and are chemically and physically stable [193]. One such medication delivery method that shields antibiotics from deterioration and can attach to biofilm constituents is lipid-based surface-functionalized PLGA [194].

Quantum dots (QDs) that are photoexcited are metallic in appearance, but they have a type of semiconductor core made of zinc or cadmium. The capacity of QDs to break down bacterial cell walls or membranes, produce free radicals, bind with genetic material, and prevent energy synthesis is what gives them their antibacterial effect. The antibacterial activity of transferrin-modified silver QDs combined with zinc and rifampicin is significantly higher than that of the zinc and rifampicin complexes [195]. The redox potential of photogenerated charge carriers that interact with the bacterial environment has been found to suppress the growth of multiple-drug-resistant (MDR) clinical isolates (*S. typhimurium*, methicillin-resistant *Staphylococcus aureus*, *Klebsiella pneumoniae*, and carbapenem-resistant *Escherichia coli*) via photoexcited QDs [196]. Factors such as the poor targeting of antibiotics to infection sites, increased dosing frequencies and side effects, the spread of resistance to currently used antibiotic medicines, the slow development rate of newer antibacterials, and the possibility of resistance to future new antimicrobial drugs all highlight the need to follow novel approaches for managing microbial infections [197].

## 5. Potential Limitations of Combatting Antibiotic Resistance Strategies

Despite the promising potential of the strategies outlined in Section 4.1, Section 4.2, Section 4.3, Section 4.4, Section 4.5, Section 4.6, Section 4.7, Section 4.8 and Section 4.9, several critical limitations impede their clinical implementation and widespread adoption. Many of these approaches remain predominantly in preclinical or early clinical investigation phases, with insufficient validation through randomized controlled trials and longitudinal studies [198]. The evolutionary adaptability of bacterial pathogens presents another significant concern, as selective pressures may drive the development of resistance mechanisms even against these novel therapies. For instance, bacteria could potentially evolve modified quorum-sensing receptors, bacteriophage resistance, or mechanisms to neutralize antimicrobial peptides from animal venoms [199]. Economic considerations further constrain practical applications, as the high costs associated with the production, purification, and quality control of biologics-based therapies (e.g., bacteriophages, stem cells, and venom-derived peptides) make them potentially inaccessible in resource-limited settings [200]. Additionally, regulatory frameworks remain underdeveloped for many of these novel approaches, creating uncertainty regarding safety assessment, standardization protocols, and approval pathways [201]. These multifaceted challenges necessitate concurrent advances in regulatory science, cost-effective manufacturing processes, and innovative clinical trial designs to facilitate the translation of promising laboratory findings into clinically viable alternatives to conventional antibiotics.

## 6. Conclusions

The antibiotic resistance crisis, fueled by the overuse of antibiotics and stagnation in new drug development, remains a critical global health challenge. This review delved into the complex resistance mechanisms of multidrug-resistant pathogens and highlighted alternative strategies, including quorum-sensing inhibitors, probiotics, antimicrobial peptides, venoms, nanobiotics, bacteriophages, CRISPR-Cas systems, immunotherapy, and photodynamic therapy. The significance of the environment as both a reservoir for resistance genes and a source of novel antimicrobials was also underscored. Despite these promising alternatives, significant challenges remain. The high costs associated with developing and commercializing new therapies, the need for rigorous regulatory approvals, and the potential for resistance evolution against novel treatments pose major hurdles. Moreover, the complexity of microbial ecosystems necessitates further research to understand the long-term efficacy and ecological impact of these interventions. Future efforts should focus on enhancing the scalability and affordability of novel antimicrobials, optimizing combination therapies to prevent resistance, and fostering global collaborations to implement sustainable antibiotic stewardship programs.

Addressing this crisis demands a unified interdisciplinary effort. Collaboration among healthcare providers, researchers, policymakers, and the public is essential to develop innovative solutions, promote awareness, and implement sustainable strategies. By advancing these approaches, we can safeguard the efficacy of existing antibiotics, ensure the development of effective treatments, and protect public health for current and future generations.

## Figures and Tables

**Table 1:** Comprehensive analysis of some bacterial antibiotic resistance mechanisms across bacterial species.

Type of Resistance	Bacterial Species	Specific Mechanism	Antibiotic Class	Resistance Strategy	Clinical Implications	References
Intrinsic	*Pseudomonas aeruginosa*	Efflux Pump Overexpression	Carbapenems	Reduced Antibiotic Accumulation	High Treatment Failure Rates	[25]
Acquired	*Staphylococcus aureus*	*mecA* Gene Horizontal	β-Lactam Antibiotics	Transfer Penicillin-Binding Protein Modification	MRSA Infections	[26]
Adaptive	*Acinetobacter baumannii*	Biofilm Formation	Multiple Antibiotics	Phenotypic Heterogeneity	Persistent Infections	[27]
Intrinsic	*Klebsiella pneumoniae*	β-Lactamase Production	Cephalosporins	Enzymatic Antibiotic Degradation	Extended-Spectrum Resistance	[28]
Acquired	*Enterococcus faecium*	Vancomycin Resistance	Gene (vanA) Glycopeptide	Antibiotics Target Site Modification	VRE Nosocomial Infections	[29]
Adaptive	Multiple Bacterial Species	Metabolic Dormancy	Broad-Spectrum	Reduced Metabolic Activity	Antibiotic Tolerance	[30]
Intrinsic	Multiple Bacterial Species	ABC Transporter Regulation	Broad-Spectrum Antibiotics	Complex Efflux Mechanism Therapeutic	Targeting Challenges	[31,32]

**Table 5:** Role of nanobiotics in defeating the challenge of antimicrobial resistance.

Advantage	Description	Ref.
Reduced Toxicity and Enhanced Stability	Encapsulating antibiotics in nanoparticles can reduce their overall toxicity and enhance their stability in vivo, preventing premature degradation.	[173]
Targeted Delivery to Sites of Infection	Nanoparticles can be designed to target specific sites of infection, either passively or actively, allowing for higher antibiotic concentrations at the infected site while minimizing systemic exposure and adverse effects.	[174]
Stimuli-Sensitive Drug Release	Nanoparticles can be engineered to release antibiotics in response to specific stimuli (e.g., pH, enzymes, reactive oxygen species) present in the infected tissues, enabling targeted and controlled drug release.	[175]
Directed towards Biofilm Microenvironments	Nanoparticles can be tailored to target and disrupt biofilms, which are a significant contributor to antimicrobial resistance, by exploiting the unique microenvironment of biofilms.	[176]
Combined Physical Therapy	Nanoparticles can be combined with other physical therapies, such as photothermal therapy (PTT) and antibacterial photodynamic therapy (aPDT), to enhance their antimicrobial efficacy through synergistic mechanisms.	[177]

## Data Availability

Data is contained in the paper.
